# Truncus arteriosus with aortic arch interruption: cardiovascular magnetic resonance findings in the unrepaired adult

**DOI:** 10.1186/1532-429X-12-16

**Published:** 2010-03-22

**Authors:** David Verhaert, Janine Arruda, Paaladinesh Thavendiranathan, Stephen C Cook, Subha V Raman

**Affiliations:** 1 The Ohio State University Medical Center, Ross Heart Hospital, Columbus, Ohio, USA; 2 Cleveland Clinic, Cleveland, Ohio, USA; 3 Nationwide Children's Hospital, Columbus, Ohio, USA

## Abstract

Truncus arteriosus (TA) is a rare congenital condition defined as a single arterial vessel arising from the heart that gives origin to the systemic, pulmonary and coronary circulations. We discuss the unique case of a 28 year-old female patient with unrepaired TA and interruption of the aortic arch who underwent cardiovascular magnetic resonance (CMR).

## Background

Truncus arteriosus communis (TA) or common arterial trunk is an uncommon congenital cardiac malformation accounting for approximately 0.7% of all complex congenital heart lesions[1]. Truncus arteriosus is defined as a single arterial vessel usually arising from both the left and the right ventricle that gives rise to the systemic, pulmonary and coronary artery circulations[2]. The truncal valve is most commonly tricuspid (70% of cases), but can be quadricuspid (21%) or bicuspid (9%) as well; occasionally more than 4 truncal leaflets have also been reported. Most commonly, patients with common trunk anomalies have a large, nonrestrictive, subarterial ventricular septal defect (VSD) situated below the truncal valve.

Collette and Edwards described four types of TA lesions based on the origin of the pulmonary arteries[3]. A Type I truncus is defined by a short main pulmonary trunk that arises from the common arterial trunk and then divides into right and left pulmonary arteries (48-68% of cases). Type II truncus is defined by the absence of a main pulmonary trunk but the left and right pulmonary vessels arise close to one another (29-48% of cases). Type III truncus also has no main pulmonary trunk and the right and left pulmonary arteries aries distant from one another (6-10% of cases). Finally, Type IV truncus is defined by the absence of pulmonary arteries and alternatively, the lungs are supplied by aortopulmonary collaterals. This form (Type IV) is no longer considered within the spectrum of TA, as it is thought to represent a variation of pulmonary atresia with VSD.

Interruption of the aortic arch (IAA) is found in approximately 11-19% of children with TA lesions [4-6]. Interruption of the aortic arch usually is defined as either type A, B or type C according to the location of discontinuity in the aortic arch[4]. Interruption of the aortic arch in TA is often accompanied by ductal continuity of the descending thoracic aorta when present[7,8]. The most common extracardiac anomaly associated with IAA is DiGeorge syndrome. Other common associated anomalies include a right aortic arch with mirror-image branching of the brachiocephalic vessels, aberrant subclavian artery, persistent left superior vena cava to coronary sinus, and secundum-type atrial septal defect.

The prognosis of patients with TA lesions is poor without surgical intervention. Newborns often present with signs of florid heart failure and cyanosis. Surgical intervention is required to avoid pulmonary vascular disease that would likely ensue in the unrepaired patient. Improving surgical techniques has led to the long-term survival of these patients[9]. Here we report the extremely unusual case of an adult patient with unrepaired TA and IAA who was evaluated by cardiovascular magnetic resonance (CMR).

## Case presentation

A 28-year old female with a history of unrepaired TA presented to the Adolescent and Young Adult Congenital Clinic for a second opinion regarding pregnancy counseling.

Initially, she reported a history of cyanosis with crying as a toddler, with subsequent diagnosis of underlying complex cyanotic congenital heart disease at age three years. Her past medical history was also significant for a cerebral abscess requiring craniotomy in 2002 with no residual neurologic defects. After this hospitalization, she was listed for heart-lung transplantation. In the interim, she had been initiated on bosentan therapy with improvement in cardiovascular symptoms. Subsequently, transplantation was not pursued. Cardiac catheterization one year later demonstrated a pulmonary artery pressure of 115/64 (mean 85 mmHg) and right atrial, pulmonary artery and arterial oxygen saturations of 44%, 84% and 78% respectively. The hemodynamics remained unchanged with administration of nitric oxide. After this procedure, sildenafil was added to her therapy with bosentan.

The patient had recently married and was actively trying to become pregnant, despite prior recommendations advising against pregnancy in light of the underlying congenital condition and severe pulmonary hypertension. Therefore, she presented for a second opinion regarding pregnancy counseling.

The patient proceeded to undergo CMR to better delineate her complex anatomy and cardiac function. A negative pregnancy test was obtained prior to scanning. Images were obtained with a 1.5 Tesla system (Magnetom Avanto, Siemens, Erlangen, Germany) with maximum gradient strength of 45 mT/m, slew rate of 200 mT/m/sec, and a 12-channel array coil with 6 anterior and 6 posterior elements. The imaging protocol included: (1) axial, sagittal and coronal half-Fourier single-shot echo train spin echo (HASTE) imaging; (2) navigator-echo acquisition covering the whole heart and great vessels; (3) steady-state free precession (SSFP) cines of the vertical long-axis, horizontal long-axis, 3-chamber, ventricular outflow views; (4) SSFP cine-stack of short-axis views from base to apex for assessment of left and right ventricular function; (5) gadolinium (0.2 mmol/kg gadopentetate dimeglumine)-enhanced 3-D T1-weighted gradient echo sequence with the slab positioned for an MRA acquisition of the common trunk, arch vessels, central pulmonary arteries and the descending aorta; (6) in- and through-plane velocity-encoded imaging of the truncal valve; and (7) late gadolinium enhancement imaging using an inversion-recovery single-shot balanced SSFP sequence with the TI optimized to null normal myocardium.

**Figure 1:**
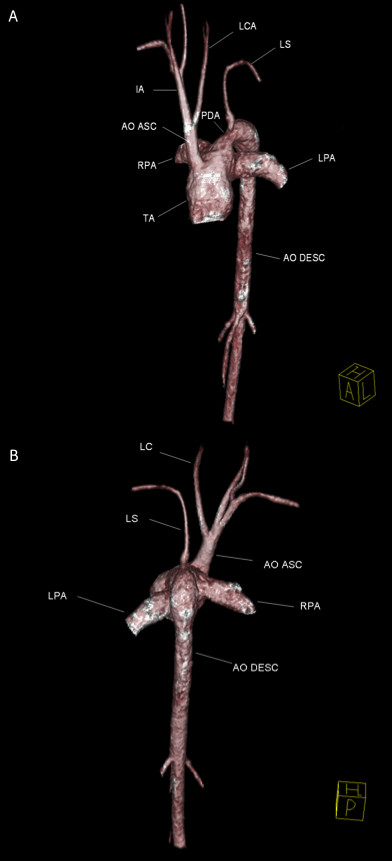
**A three-dimensional volume-rendered magnetic resonance angiogram; anterolateral (A) and posterior (B) views demonstrate anatomy consistent with Type II truncus arteriosus and interruption of the aortic arch (Type B)**. TA: truncus arteriosus; Ao Asc: ascending aorta; Ao Desc: descending aorta; RPA: right pulmonary artery; LPA: left pulmonary artery; LCA: left common carotid artery; LS: left subclavian artery; PDA: patent ductus arteriosus; IA: innominate artery

**Figure 2:**
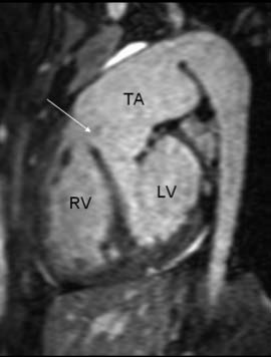
**An oblique sagittal reconstruction from the whole heart navigator showing the large, subarterial ventricular septal defect (arrow)**. LV: left ventricle; RV: right ventricle

**Figure 3:**
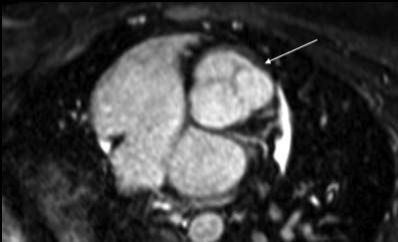
**An oblique axial reconstrunction from the whole heart navigator demonstrating the trileaflet appearance of the truncal valve (arrow)**.

**Figure 4:**
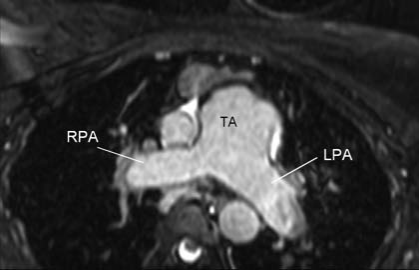
**An oblique axial image demonstrates widely patent branch pulmonary arteries**. LPA: left pulmonary artery; RPA: right pulmonary artery; TA: truncus arteriosus.

The 3-D MRA sequence demonstrated a dilated common arterial trunk with the left and right pulmonary arteries arising from a short main pulmonary trunk at the posterior side of the common arterial trunk (Figure 1A-B). The ascending aorta appeared hypoplastic (1.2 × 1.3 cm) with IAA between the left common carotid artery and the left subclavian artery (Type B). Perfusion to the descending aorta was maintained by a large patent ductus arteriosus. The left ventricle (LV) was normal in size (end-diastolic volume 68 mL/m^2^), with LV ejection fraction of 52%. The right ventricle (RV) was also normal in size (72 mL/m^2^) with moderate concentric hypertrophy and RV ejection fraction 51%. Systolic flattening of the interventricular septum was present suggesting right ventricular pressure overload (see Additional file 1). A large, subarterial VSD was noted beneath the truncal valve (Figure 2). The truncal valve was trileaflet with mild thickening of the leaflets but without stenosis or insufficiency (Figure 3). There was no evidence of branch pulmonary artery stenosis which may occasionally protect unrepaired TA patients from developing irreversible pulmonary vascular disease (Figure 4). Findings supporting the presence of an interruption included the descending aorta arising from the ductus, or ductal arch, with the left subclavian artery arising from the proximal descending aorta. There also appeared to be an absence of arch continuity between the left common carotid left subclavian arteries. Lastly, the inverse relationship between the small size of the ascending aorta and the large patent ductus often provided a final clue that the arch was interrupted[10].

Based on CMR, this patient's conotruncal anomaly could best be classified as type I B - a very uncommon form, accounting for only 3% of all TA lesions[5]. Reports of adults with TA without surgical palliation remain extraordinarily uncommon. This is the first report of a patient with this type of truncal anatomy who survived into adulthood without surgical repair. The unoperated natural history of TA patients is extremely poor, with death in infancy or early childhood due to severe heart failure, subsequent pulmonary vascular occlusive disease, endocarditis and cerebral abscess. The fact that the truncal valve in our patient was trileaflet and competent may have played an important role in her survival, since it is well known that truncal valve insufficiency is associated with higher rates of early and late mortality[9].

Although pregnancy and delivery have been reported in the patient who has undergone complete repair of TA, with regards to pregnancy counseling, Eisenmenger physiology is an absolute contraindication to pregnancy. Maternal mortality is reported as high as 50%[11]. Furthermore, the patient was counseled regarding the teratogenic risks of her current medical regiment that includes bosentan (FDA: category X). She also received genetic counseling regarding the increased risk of congenital heart disease as well as DiGeorge syndrome (50%). The patient nevertheless became pregnant shortly after consultation, which resulted in spontaneous abortion several weeks later.

Our case illustrates the important role of CMR in characterizing and defining complex anatomy in the adult with congenital heart disease. The information provided by this CMR examination allowed the Adult Congenital Team to counsel this patient regarding pregnancy and contraception. The ability of CMR to depict morphology and altered hemodynamics in the adult with congenital heart disease has rapidly made this imaging modality indispensible in the care of this patient population.

## Consent

Written informed consent was obtained from the patient for publication of this case report and any accompanying images.

## Competing interests

Dr. Raman receives research support from Siemens.

## Authors' contributions

DV, JA and SVR were involved in data acquisition, analysis and manuscript preparation; DV and PT were involved in the preparation of the manuscript and accompanying images; and SCC provided critical review of the manuscript.

## Supplementary Material

Additional file 1 **Supplementary movie file 1**. Stack of short-axis SSFP cines, demonstrating preserved biventricular systolic function. The presence of right ventricular (RV) hypertrophy and systolic flattening of the interventricular septum suggest evidence of RV pressure overload. Click here for file
